# Supplementary material for: The Effect of Floor Height on Secondhand Smoke Transfer in Multiunit Housing
Source: Int J Environ Res Public Health. 2022 Mar 23;19(7):3794. doi: 10.3390/ijerph19073794 (PMC8997625; doi:10.3390/ijerph19073794)
Supplement: Supplementary file 1 [file ijerph-19-03794-s001.zip › ijerph-1623125-supplementary.pdf]

Table S1. Descriptive Statistics<br>Nicotine Concentration, 21 Low-Income High-Rise Buildings – Aggregated by Season				
**Apartments**				
	**N**	**M (SD) or %**	**95% CI**	**p-value**
**Floor < 7**	199			
Geometric Mean		0.023 (2.29)	[0.020-0.025]	0.308
% above LOD		15.1		0.146
**Floor 8-14**	281			
Geometric Mean		0.023 (2.17)	[0.021-0.026]	
% above LOD		17.1		
**Floor > 15**	386			
Geometric Mean		0.025 (2.27)	[0.023-0.027]	
% above LOD		21.2		
**Stairwells**				
	**N**	**M (SD) or %**	**95% CI**	**p-value**
**Floor < 7**	21			
Geometric Mean		0.15 (4.53)	[0.07-0.29]	0.003
% above LOD		76.2		0.031
**Floor 8-14**	75			
Geometric Mean		0.23 (4.85)	[0.16-0.33]	
% above LOD		82.7		
**Floor > 15**	62			
Geometric Mean		0.45 (3.30)	[0.33-0.60]	
% above LOD		95.2		
**Hallways**				
	**N**	**M (SD) or %**	**95% CI**	**p-value**
**Floor < 7**	46			
Geometric Mean		0.18 (4.98)	[0.11-0.28]	0.962
% above LOD		78.3		0.643
**Floor 8-14**	68			
Geometric Mean		0.16 (4.18)	[0.12-0.23]	
% above LOD		77.9		
**Floor > 15**	60			
Geometric Mean		0.16 (5.58)	[0.11-0.26]	
% above LOD		71.7		
